# Chemopreventive potential of β-Sitosterol in experimental colon cancer model - an *In vitro *and *In vivo *study

**DOI:** 10.1186/1472-6882-10-24

**Published:** 2010-06-04

**Authors:** Albert A Baskar, Savarimuthu Ignacimuthu, Gabriel M Paulraj, Khalid S Al Numair

**Affiliations:** 1 Division of Ethnopharmacology, Entomology Research Institute, Loyola College, Chennai - 600 034, Tamil Nadu, India; 2 Department of Community Health Sciences, College of Applied Medical Sciences, King Saud University, P.O. Box 10219, Riyadh, 11433, Kingdom of Saudi Arabia

## Abstract

**Background:**

*Asclepias curassavica *Linn. is a traditional medicinal plant used by tribal people in the western ghats, India, to treat piles, gonorrhoea, roundworm infestation and abdominal tumours. We have determined the protective effect of β-sitosterol isolated from *A. curassavica *in colon cancer, using *in vitro *and *in vivo *models.

**Methods:**

The active molecule was isolated, based upon bioassay guided fractionation, and identified as β-sitosterol on spectral evidence. The ability to induce apoptosis was determined by its *in vitro *antiradical activity, cytotoxic studies using human colon adenocarcinoma and normal monkey kidney cell lines, and the expression of β-catenin and proliferating cell nuclear antigen (PCNA) in human colon cancer cell lines (COLO 320 DM). The chemopreventive potential of β-sitosterol in colon carcinogenesis was assessed by injecting 1,2-dimethylhydrazine (DMH, 20 mg/kg b.w.) into male Wistar rats and supplementing this with β-sitosterol throughout the experimental period of 16 weeks at 5, 10, and 20 mg/kg b.w.

**Results:**

β-sitosterol induced significant dose-dependent growth inhibition of COLO 320 DM cells (IC_50 _266.2 μM), induced apoptosis by scavenging reactive oxygen species, and suppressed the expression of β-catenin and PCNA antigens in human colon cancer cells. β-sitosterol supplementation reduced the number of aberrant crypt and crypt multiplicity in DMH-initiated rats in a dose-dependent manner with no toxic effects.

**Conclusion:**

We found doses of 10-20 mg/kg b.w. β-sitosterol to be effective for future *in vivo *studies. β-sitosterol had chemopreventive potential by virtue of its radical quenching ability *in vitro*, with minimal toxicity to normal cells. It also attenuated β-catenin and PCNA expression, making it a potential anticancer drug for colon carcinogenesis.

## Background

Lung, breast and colon cancer are the 3 most common cancers worldwide, with an increasing annual incidence [1]. Scavenging reactive oxygen species (ROS) by antioxidant activity is important in preventing potential damage to cellular components, including DNA, proteins, and lipids. Oxidative damage can cause major events, sometimes leading to carcinogenesis. Antioxidants have been used to inhibit apoptosis because apoptosis appeared initially to be mediated by oxidative stress [2]. Several *in vitro *and *in vivo *studies suggest that plant sterols can protect against colon, prostate, and breast cancers in developed countries [3]. Colon cancer is frequently a pathological consequence of persistent oxidative stress, leading to DNA damage and mutations in cancer-related genes, cell cycle death and regeneration, where cellular overexpression of β-catenin and proliferating cell nuclear antigen (PCNA) are implicated [4]. Increase in the cytoplasmic pool of β-catenin is associated with cell proliferation, and resistance to apoptosis and carcinogenesis [5].

1,2-Dimethylhydrazine (DMH) induced colon carcinogenesis in the rat is a widely used experimental model among cancer chemoprevention studies. Aberrant crypt foci (ACF) are putative preneoplastic lesions of colonic neoplasia in rodents and humans. During the process of colon carcinogenesis, ACF appear in the early stages and subsequently develop into polyps, adenomas and eventually carcinomas [6]. The goal of cancer chemoprevention is to retard, block or reverse the process of carcinogenesis through the use of natural or synthetic agents, including antioxidants. *A. curassavica *powder is used to treat abdominal tumors in traditional Indian medicine. β-sitosterol and its glycosides, along with other compounds such as oleanolic acid, uzarigenin, calactin, calotropin, coroglaucigenin, calotropagenin and uzarin have been isolated. The cytotoxic principle of *A. curassavica *proved to be calotropin [7].

β-sitosterol isolated from various plants promotes apoptosis by increasing FAS levels and caspase-8 activity [8], phosphorylation of extracellular-signal regulating kinase (ERK) and p38 mitogen-activated protein kinase (MAPK) [9], inhibition of cancer cell proliferation - even at low concentrations with no cytotoxic effect to noncancerous cells [10], modulation of antioxidant enzyme levels in pathogenesis [11], arrest of cells at G2/M phase in prostate cancer cells [12], and decreasing free radical generation *in vitro *[13]. A large number of medicinal plants and their purified constituents have beneficial therapeutic potentials. In this context, our work aimed to broaden the understanding of the anticancer potential of β-sitosterol in *in vitro *cancer model and DMH-induced experimental colon carcinogenesis model, which could be beneficial in anticancer therapy.

## Methods

### Chemicals

Dulbecco's modified Eagle's medium (DMEM), Rosewell Park Memorial !nstitute 1640 medium (RPMI-1640), 2,2-diphenyl-1-picrylhydrazyl (DPPH), 3-(4,5-dimethyl thiazol-2-yl)-2,5-diphenyl tetrazolium bromide (MTT), 2',7'-dichlorofluorescein (DCF-DA), HOECHST 33258, and 1,2-dimethylhydrazine (DMH) were purchased from Sigma Chemical Company. Fetal bovine serum (FBS), phosphate buffered saline, Trypsin-EDTA - 0.25%, Antibiotic-antimycotic solution from Invitrogen (USA). AnnexinV-FITC antibody and propidium iodide kit were purchased from Biosource, Camarillo, CA USA. The commercial rat pellet diet used was purchased from Hindustan Lever Ltd, Mumbai, India. All other chemicals, including solvents, were of high purity analytical grade marketed by HiMedia chemicals, Mumbai, India.

### Isolation of β-sitosterol from A. curassavica

*A. curassavica *leaves were collected from Kodhaiyar, Western Ghats, Tamil Nadu and carefully identified by Dr. Ayyanar, a taxonomist at the Department of Botany, Loyola College, Chennai, India. One kilogram of shade dried leaves was placed in an aspirator; 3 L hexane were added and the mixture shaken occasionally for 48 h. Extracts were filtered through Whatman filter paper no. 2 on a Buchner funnel and the solvent removed under reduced pressure in a rotary evaporator at 40°C. The extracts were placed in preweighed flasks before drying. The remaining plant residue was extracted sequentially with ethyl acetate and methanol. The extracts obtained were dissolved in dimethyl sulphoxide (DMSO) and used as a stock solution. This stock solution was filter-sterilized (0.22 μm) prior to experimental use.

Active ethyl acetate extract (20 g) was subjected to column chromatography and eluted with hexane, followed by combinations of hexane:ethyl acetate ranging from 95:5 to 0:100. The eluted fractions were combined to give major fractions by comparing the R_f _values of the collected fractions when run on TLC F_254 _plates in similar solvent systems. Based upon the TLC pattern, the fractions were pooled to give 17 major fractions. The collected fractions were screened for their anticancer property, based on bioassay guided fractionation. F12 was identified as active. White crystals deposited on the walls after further purification of F12. These crystals produced a single spot on TLC, confirmed to be a single compound, which was subjected to IR, MASS, and NMR analysis for structure elucidation. The physical and chemical data of F12 corresponded to that of β-sitosterol. The crude, fractions and the isolated compound were screened for cytotoxicity using human colon cancer cell line - COLO 320 DM, human gastric cancer - AGS, human breast cancer - MCF-7 and human liver cancer - A549, and a normal monkey kidney - VERO. The extracts, fractions and β-sitosterol showed promising antiproliferative activity in COLO 320 DM cells (preliminary data - not shown).

### *In vitro *antioxidant activity

DPPH free radical scavenging assays were carried by the method of Blois [14]. Antioxidants react with stable free radical DPPH and are converted into 1,1-diphenyl-2-picryl-hydrazine. The ability to scavenge DPPH was measured by the decrease in absorbance at 517 nm, using ascorbic acid as a standard antioxidant for comparison. Nitric oxide (NO) was generated from sodium nitropruside and measured by Griess' reaction as described by Garrat [15].

### Cell lines

Human COLO 320 DM and monkey VERO cell lines purchased from the National Center for Cell Science (NCCS, Pune) were cultured in 75-cm^2 ^flasks containing DMEM for VERO and RPMI-1640 for the COLO 320 DM line. Both media were supplemented with 10% FBS, 10,000 U/ml penicillin, 10 mg/ml streptomycin, and 25 μg/ml ampotericin B. Cells were cultured as monolayers in culture flasks at 37°C at 95% humidity in 5% CO_2 _in air. After reaching 80-90% confluence, cells were trypsinized and subcultured. During the experiments, serum-containing medium was replaced by serum-free medium containing 15, 30, 60, 120 and 240 μM/ml β-sitosterol dissolved in DMSO and the stock maintained at -20°C. The final working concentration of DMSO was <0.1%.

### Antiproliferative activity

Survival of cells was assayed by the MTT method of Mosmann [16] after treatment with 15, 30, 60, 120 and 240 μM/ml of compound for 24 h. Optical density (OD) was measured using a 96-well micro plate-reader (BIO-RAD, model 680, USA) at 570 nm.

### Flow cytometric analysis

Apoptosis was determined using FITC-labeled AnnexinV antibody by flow cytometry [17]. The fraction of the cell population in different quadrants was analyzed using quadrant statistics. Cells in the lower right quadrant represented apoptosis, and those in the upper right quadrant represented necrosis or post-apoptotic necrosis [18].

### Assessment of ROS levels

COLO 320 DM cells were incubated with 5 μM DCF-DA for 20 min and washed 3 times with PBS. Levels of intracellular ROS were subsequently determined by image analysis of DCF-DA loaded cells with a confocal microscope (excitation at 488 nm and emission at 525 nm). DCF-DA is membrane permeable and interacts with reactive oxygen species in living cells, emitting light in the green wavelength.

### DNA fragmentation

To confirm morphological changes in the nuclei, cells were seeded in 16-mm cover slips placed in 6-well plates at 2 × 10^6 ^cells. Cells were treated 1 day after seeding with 15, 60 and 120 μM β-sitosterol for 24 h. HOECHST 33258 solution was added (20 μg/mL), and the cells incubated for 30 min before being examined by fluorescence microscopy (Olympus, Tokyo, Japan).

### Expression of PCNA and β catenin

Fifty μg protein of the total cell lysate was mixed with an equal volume of 2× sample buffer (125 mM Tris-HCl (pH 6.8), 4% SDS, 20% glycerol, 10% β-mercaptoethanol and 0.004% bromophenol blue), boiled for 5 min at 95°C, cooled, loaded on each lane of 8-15% polyacrylamide gels, and separated by SDS-PAGE at room temperature. The resolved proteins were electrophoretically transferred to nitrocellulose membranes. The membranes were blocked in 5% non-fat milk in Tris-buffered saline with 0.1% Tween 20 for 1 h at room temperature, and probed with β-catenin [mouse monoclonal antibody dilutioned 1:1000] (BD transduction laboratories, USA); PCNA [mouse monoclonal antibody diluted 1:1000] (Santa Cruz biotechnology, USA) primary antibodies overnight at 4°C. The blots were extensively washed with Tris-buffered saline with 0.1% Tween 20 and incubated with respective anti-mouse HRP labeled secondary antibody at a dilution of 1:2000 for 1 h at room temperature. After extensive washes in TBS-T, bands were visualized by treating the membranes with 3,3'-diaminobenzidine tetrahydrochloride. The membranes were photographed and the bands quantified with image analysis software (Imagej, NIH, USA). Densitometry data presented in the bar graphs are the "fold change" compared with control in each case.

### Animals

Male albino Wistar rats aged 5 weeks obtained from Central Animal House, Kings Institute, Chennai, Tamil Nadu, were used. The animals were cared for in compliance with the principles and guidelines of Ethical Committee for Animal Care and institutional animal ethical committee, in accordance with the Indian National Law on Animal Care and Use (Reg. No. 833/a/2 004/CPC SEA). The animals were housed 4 per polypropylene cage with a wire-mesh top and a hygenic bed of husk in a specific pathogen-free animal room under controlled conditions of a 12 h light/12 h dark cycle with temperature of 24 ± 2°C and relative humidity of 50 ± 10% until the end of the experimental period. The rats were held in quarantine for 1 week and had access to food and tap water *ad libitum*. Commercial pellet diet containing 4.2% fat was powdered and mixed with 15.8% peanut oil, making a total of 20% fat. This modified diet was fed to all rats throughout the 16 week experimental period.

### Administration of carcinogen

The experimental animals were divided into 6 groups. Initial body weights of all animals in the protocol were between 80-120 g. Animal weights were recorded once a week throughout the experimental period, and prior to sacrifice.

The animals in groups 3 - 6 received subcutaneous injections of DMH at 20 mg/kg b.w. once a week for the first 4 consecutive weeks. Prior to subcutaneous injection, DMH was dissolved in 1 mM EDTA; the pH was adjusted to 6.5 with 1 mM NaOH to ensure the pH and stability of the chemical, and used immediately after preparation.

#### β-sitosterol treatment of the animals

Group 1 - Rats received modified pellet diet along with intragastric intubation of 0.1% carboxymethyl cellulose (CMC) (1.0 mL), throughout the experimental period.

Group 2 - Rats received modified pellet diet + 20 mg/kg b.w. β-sitosterol suspended in 0.1% CMC (1.0 mL), p.o., everyday throughout the experimental period.

Group 3 - Rats were administered with 20 mg/kg b.w. DMH (carcinogen) s.c. once a week for 4 consecutive weeks and no further treatment for the next 12 weeks.

Group 4 - The animals were treated as in group 3 along with β-sitosterol (5 mg/kg b.w., p.o.) supplemented throughout the entire experimental period of 16 weeks.

Group 5 - The animals were treated as in group 3 along with β-sitosterol (10 mg/kg b.w., p.o.) supplemented throughout the entire experimental period of 16 weeks.

Group 6 - The animals were treated as in group 3 along with β-sitosterol (20 mg/kg b.w., p.o.) supplemented throughout the entire experimental period of 16 weeks.

At the end of the experiment, colons were processed to assess ACF by the method of Bird [19]. The total number of ACF/rat was calculated from the sum of all ACF. To determine crypt multiplicity, the number of aberrant crypts in each focus was recorded.

### Statistical analysis

The results of the cytotoxicity tests (n = 6) were calculated as the percentage growth inhibition relative to the control. IC_50 _values for growth inhibition were derived from a non-linear regression model (curvefit), based on sigmoidal dose-response curve (variable), computed using GraphPadPrism (Graphpad). Data are given as mean ± S.E.M. The statistical significance was set at P < 0.05. Statistical evaluation involved one-way analysis of variance (ANOVA) followed by Duncan's multiple range test (DMRT).

## Results

**Figure 1:**
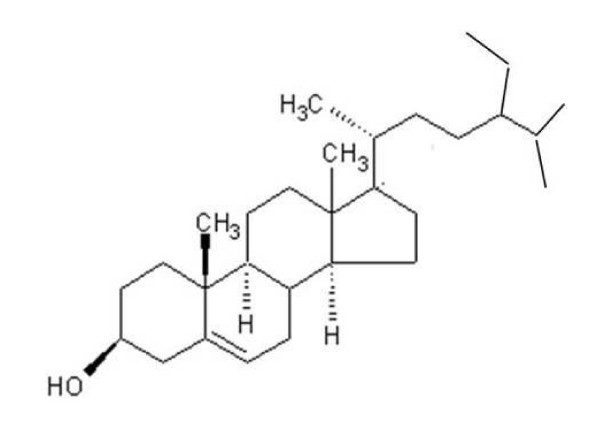
**Structure of β-sitosterol isolated from *A. curassavica***. The compound was identified based on the following evidence: IR spectrum showed hydroxyl (3430 cm^-1^) and tris-substituted double-bond (1642 and 80.1 cm^-1^). The EI/MS mass spectrum showed m+ at m/z 414, corresponding to the molecular formula (C_29_H_50_O) for β-sitosterol. The other characteristic peaks were at m/z 273 [m-sidechain]^+ ^255 [m-side chain - H_2_O]^+ ^231 [m-side + +1chain-ring D], 213 [231-H_2_O] and 300. The H-NMR spectrum showed 2 tertiary methyl groups at δ 0.68 and 1.02, corresponding to H-18 and H-19. Three secondary methyls appeared at δ 0.92, 0.82 and 0.84, corresponding to H-21, H-26 and H-27, respectively. (J = 6.5 Hz). H-29 appeared as triplet at δ 0.85 (J = 7.0 Hz). The C-NMR spectrum indicated 29 carbon atoms, 10 primary, 10 secondary and 3 tertiary carbon; 6 methyl groups being present. C-5 and C-6 were olefinic carbon atoms appearing at δ 121.70 and 140.74.

**Figure 2:**
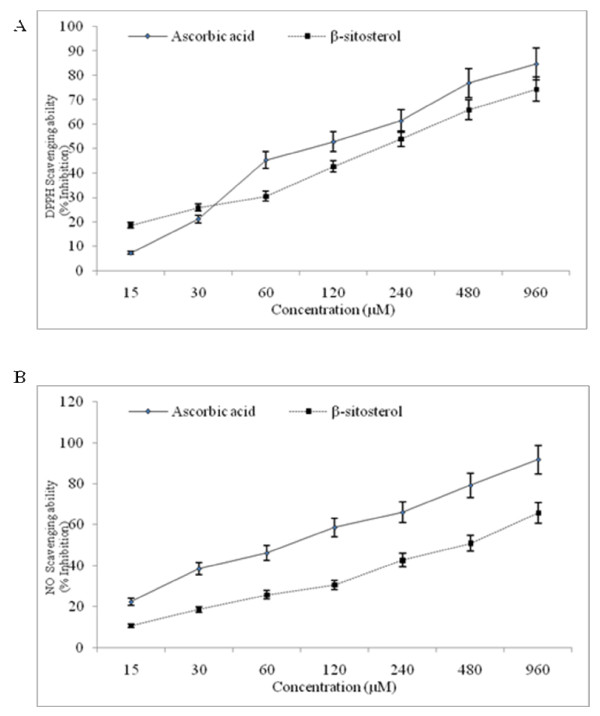
**Anti-oxidant activity of β-sitosterol**. The free radical scavenging ability of β-sitosterol was determined by DPPH and nitric oxide scavenging assays compared with ascorbic acid as a standard at various concentrations.

**Figure 3:**
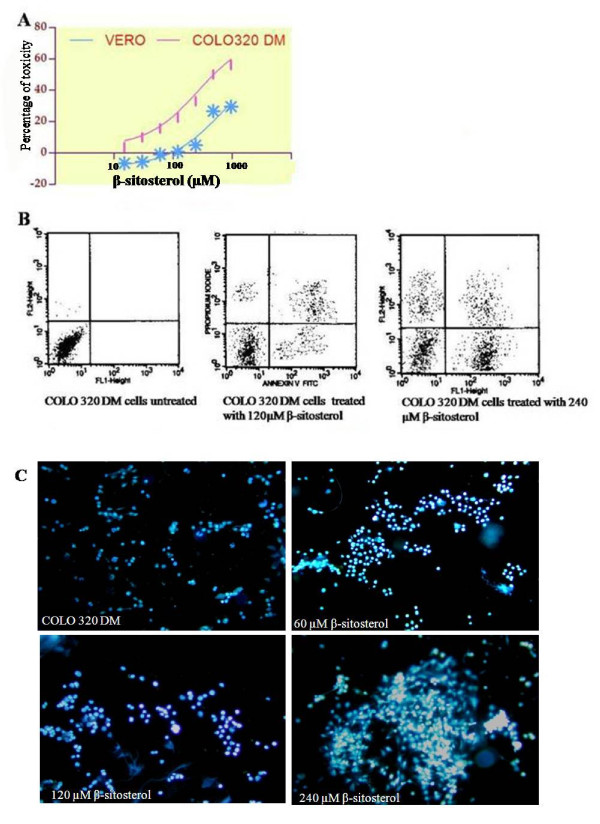
**Induction of apoptosis in COLO 320 DM**. Human colon cancer cell lines (COLO 320 DM) cells were treated 120 or 240 μM β-sitosterol. The anti-proliferative effect is shown in (a), flow cytometric analysis for the determination of pro-apoptotic/apoptotic/necrotic cells is given in (b), and fluorescent staining of nuclei by Hoechst 33258 at 20× is seen in (c).

The purified compound isolated from *A. curassavica *was identified as β-sitosterol based on Physiochemical evidence (Figure 1) as by extraction method listed in "Materials and Methods". The total collected ethyl acetate extract was 2.2 kg, and the yield of β-sitosterol 1.85 gm. β-sitosterol had anti-radical activity, with an IC_50 _of 389.5 μM and 448.2 μM for DPPH and NO scavenging assays, respectively (Figure 2A &2B). β-sitosterol was noticeably inhibitory within 24 h at 30 μM and significantly more so at 240 μM (P < 0.05; Figure 3A), with minimal toxicity in non-cancerous cells (VERO). IC_50 _values were 266.2 μM for COLO 320 cells and >1 mM for VERO cells.

### Induction of apoptosis, and PCNA and β-catenin expression by β-sitosterol

An increased number of COLO 320 DM cells treated with 240 μM β-sitosterol stained with Annexin FITC^+^/PI^+^, with a lower percentage of necrotic or propidium iodide stained cells after 24 h incubation (Figure 3B). β-sitosterol induced DNA fragmentation even at a 15 μM; increased DNA fragmentation and DNA tailing also ocurred in cancer cells treated with 120 μM β-sitosterol (Figure 3C).

**Figure 4:**
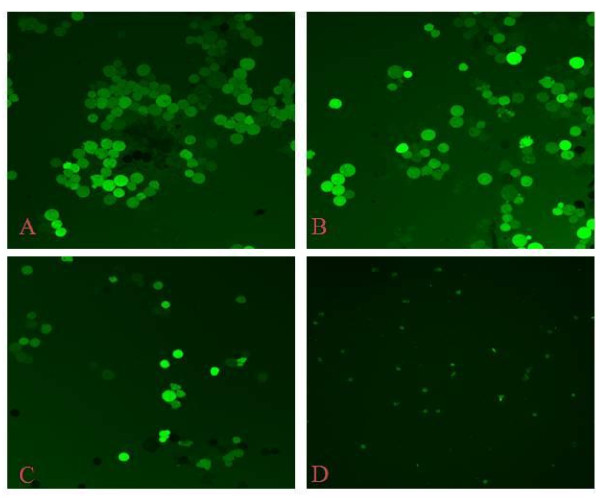
**ROS Scavenging ability**. β-sitosterol was treated with COLO 320 DM cells for 24 h at 120 or 240 μM and the ROS scavenging ability determined using DCF staining and confocal microscopy. (A & B) COLO 320 DM cells untreated, (C) COLO 320 DM cells treated with β-sitosterol at 120 μM, (D) COLO 320 DM cells treated with β-sitosterol at 240 μM.

DCF fluorescence in COLO 320 DM cells indicates enhanced free radical generation in living cells (Figure 4A). After treatment with β-sitosterol for 24 h, fluorescence clearly reduced (Figure 4D).

**Figure 5:**
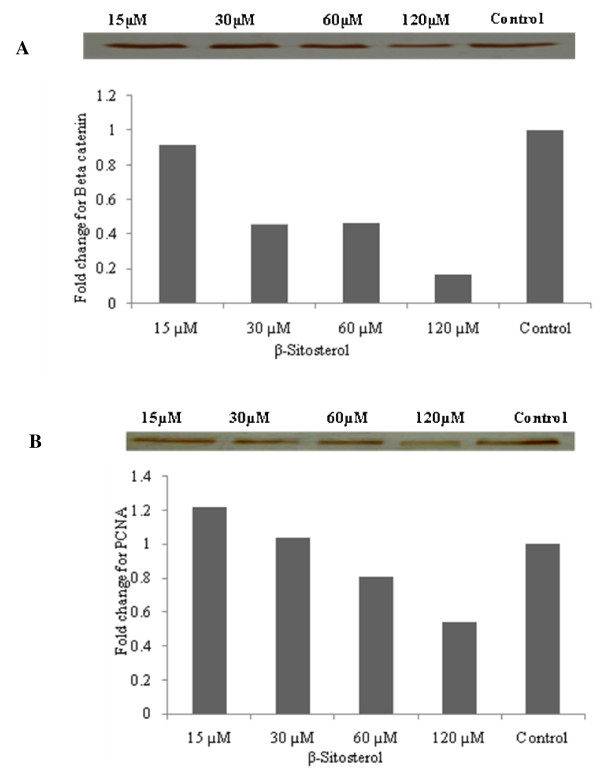
**Expression of β catenin (A) and PCNA (B) in COLO 320 DM cells**. Human colon cancer (COLO 320 DM) cells were treated with 15, 30, 60, or 120 μM/ml β-sitosterol for 24 h and 50 μg of extracted protein were loaded.

Colonic cell proliferation and β-catenin expression were analyzed using human colon cancer (COLO 320 DM) cells treated with β-sitosterol for 24 h. β-sitosterol administration significantly decreased β-catenin and PCNA expression in COLO 320 DM cells *in vitro *(Figure 5).

### Inhibition of ACF development by β-sitosterol

**Figure 6:**
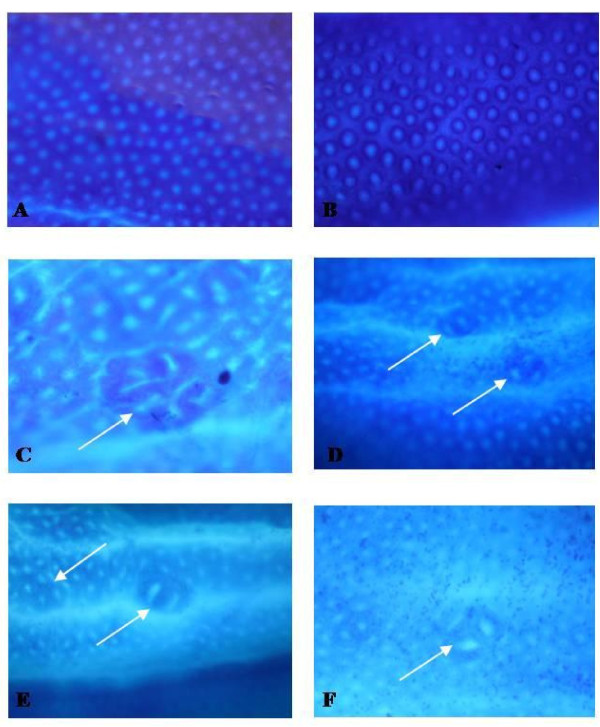
**Topographical view of ACF**. Topographical views of (A & B) normal crypt (40×), (C) ACF (arrows) with multiple crypts in the colon from a rat treated with DMH (40×), (D) ACF (arrow) with 2 and 3 crypts in the colon from a rat treated with DMH+ β-sitosterol (5 mg/kg), (E) ACF (arrow) with multiple 2 crypts in the colon from a rat treated with DMH+ β-sitosterol (10 mg/kg), and (F) ACF (arrow) with single 2 crypts in the colon from a rat treated with DMH+ β-sitosterol (20 mg/kg).

β-sitosterol supplementation *per se *did not induce aberrant crypt formation in colonic mucosa of non-DMH treated rats. β-sitosterol treatment for 16 weeks significantly also reduced the number of aberrant crypt and crypt multiplicity in a dose-dependent manner in DMH-treated rats (Table 1, Figure 6).

**Table 1:** Effect of β-sitosterol on development of ACF induced by DMH

	ACF formation in rat colon			
	
Groups	Total no. of ACF	No. of AC	Crypt multiplicity (AC/ACF)	% of ACF inhibition
DMH	52 ± 5.23^a^	151 ± 12.31^a^	2.9 ± 0.31^a^	-
DMH + β-sitosterol (5 mg/Kg)	47 ± 4.68^a^	126 ± 10.36^b^	2.6 ± 0.25^b^	38.2
DMH + β-sitosterol (10 mg/Kg)	38 ± 4.12^b^	92 ± 10.21^c^	2.4 ± 0.18^c^	52.8
DMH + β-sitosterol (20 mg/Kg)	29 ± 3.14^c^	54 ± 8.94^d^	1.8 ± 0.16^d^	73.6

Each value is mean ± SD for 6 replicates in each group. Values carrying different letters in a row differ significantly at p < 0.05 by DMRT.

## Discussion

Elevated oxidative stress can modify a number of cellular targets and cause cell damage, and the subsequent lack of repair has been considered responsible for carcinogenesis [20]. Cancer cells can be subject to increased and persistent oxidative stress due to elevated levels of intracellular ROS generation. Reducing oxidative stress can therefore suppress the proliferation of tumor cells and enhance apoptosis [21]. Natural anti-oxidants have a wide range of biochemical activities, including inhibition of ROS generation, direct or indirect scavenging of free radicals, and alteration of anti-oxidant potential [22]. Many anti-oxidants have been used to inhibit apoptosis that is probably mediated by oxidative stress. Many anti-oxidant substances have anti-cancer or anti-carcinogenic properties [23]. A variety of cytotoxic/anti-cancer drugs induce apoptosis in malignant cells *in vitro *[24]. Use of anti-neoplastic drugs needs to be based on their *selective *toxicity to malignant cells, their advantage being this selectivity. Ideally, this goal is to have with little or no effect on normal cells, the major problem faced in other cancer therapies, many of which now combine a variety of modalities to reduce this problem. Medicinal plants are mostly edible and do not exert toxic effects when taken regularly. They exhibit anti-cancer potential by scavenging nitric oxide radicals [25]. *In vitro *screening models provide important preliminary data to select drugs with potential anti-neoplastic properties for preclinical and clinical trials.

β-sitosterol can scavenge the nitric oxide radicals generated *in vitro *by DPPH and NO scavenging assays by donating their hydrogen atom to quench the free radicals, indicating that β-sitosterol is a potential anti-oxidant. Cytotoxicity screening models provide important preliminary data to select compounds with potential antineoplastic properties for future work. A variety of cytotoxic drugs can induce apoptosis of malignant cells *in vitro *[24]. β-sitosterol showed cancer cell specific cytotoxic effects by inhibiting proliferation of COLO 320 DM cells, while showing little toxicity in VERO cells. β-sitosterol has been reported to possess cytotoxic effects in breast cancer and Bowes cell lines [8,26]. The present study show β-sitosterol inhibits proliferation of colon cancer cells with less toxicity towards normal cells *in vitro*.

β-catenin and PCNA are useful markers of proliferative activity in colon carcinogenesis [27-29]. β-sitosterol administration significantly decreased the expression of β-catenin and PCNA (Figure 5). Oxidative stress augments Wnt-β catenin signalling machinery and PCNA [30]. β-catenin plays a critical role as a component of the cell adhesion complex and activated β-catenin signaling favors cellular proliferation as well as exerting an anti-apoptotic effect on a variety of cancers [31]. Positive correlation between β-catenin accumulation and cell proliferation in colon carcinoma has been reported [29]. Furthermore, excess β catenin promotes accumulation of transcriptionally active p53 that may favor survival rather than apoptosis [32]. While β-sitosterol posses anti-proliferative and apoptotic potential in several cancer models [33], the mechanism of action is not exactly known. PCNA is a DNA polymerase δ auxiliary protein that accumulates in the nuclei during late G1 and early S phases. The fraction of PCNA-expressing cells is used as an indicator of DNA synthesis and cellular proliferation [34]. Oxidative stress in cancer cells is maintained at high levels that promote cellular proliferation [35]. Treatment with β-sitosterol markedly decreased the proliferative index as revealed by the down regulation of β-catenin and PCNA expression in COLO 320 DM cells. Antioxidants have been reported to suppress the β-catenin mutation in hepatocellular carcinomas in rats [36]. Hence this ability of β-sitosterol to suppress the levels of β-catenin, PCNA and its target genes might be attributed to its antioxidant property. β-sitosterol suppresses the altered β-catenin and PCNA mutation in COLO 320 DM cells and pushes the cells towards apoptosis by their ability to scavenge the radicals produced in COLO 320 DM cells.

ACF are putative preneoplastic lesions in colon carcinogenesis and considered as surrogate precursors lesions of colorectal cancer. ACFs are readily discernible morphological changes within the colonic mucosa of rodents that may contribute to the stepwise progression of colon cancer [37]. Natural compounds that inhibit ACF induced by colon carcinogens are protective against colon cancer in rodents [38]. β-sitosterol supplementation decreased the number of ACFs in DMH-induced animals in a dose-dependent manner. PCNA and β-catenin levels are increased in animals treated with DMH and treatment with an anti-cancer compound reduces the incidence of ACFs in DMH-induced animals [31]. The levels of β-catenin and PCNA are directly proportionate to ACF development in colon carcinogenesis. The anti-carcinogenic property of β-sitosterol in DMH-induced colon carcinogenesis is due to its anti-oxidant properties. The ability to suppress the β-catenin mutation in DMH-induced animals in short-term colon carcinogenesis suggests that β-sitosterol may have a chemopreventive effect on the development of tumors upon longer treatment protocols. β-sitosterol supplementation at 20 mg/kg b.w. significantly reduced the incidence of ACF in carcinogen treated animals by 73.6%. Hence from both our *in vitro *and *in vivo *results it is evident that β-sitosterol exhibits chemopreventive potential in DMH induced experimental carcinogenesis by inhibiting levels of β-catenin and PCNA accumulation in colon cancer. The present study confirms the anti-carcinogenic potential of β-sitosterol and the effective dose to be 20 mg/kg b.w. in 1, 2-dimethylhydrazine induced colon carcinogenesis

## Conclusions

The anticarcinogenic property of β-sitosterol in DMH-induced colon carcinogenesis is due to its antioxidant property and its ability to suppress the altered β-catenin and PCNA expression in colonic mucosa of DMH-treated rats in short-term colon carcinogenesis. Hence the study suggests that β-sitosterol exerts a chemopreventive effect in DMH-induced experimental carcinogenesis, indicating its potential as an anticancer drug. The effective dose for long-term studies will be 10-20 mg/kg b.w. in experimentally induced colon carcinogenesis.

## Competing interests

The authors declare that they have no competing interests.

## Authors' contributions

AB and SI designed the study and wrote the manuscript, GM and KN collated all statistical information. AB and GM involved in data collection and interpretation. SI and KN coordinated and oversaw the study. All authors read and approved the final manuscript.

## Pre-publication history

The pre-publication history for this paper can be accessed here:

http://www.biomedcentral.com/1472-6882/10/24/prepub
